# An Arterial Anastomosis Stenosis Used in Lieu of Banding to Prevent Dialysis Access Steal Syndrome

**DOI:** 10.7759/cureus.24757

**Published:** 2022-05-05

**Authors:** Juliana N Young, Cynthia A Reyes, Ayodele O Erinle

**Affiliations:** 1 Medicine, Burrell College of Osteopathic Medicine, Las Cruces, USA; 2 Pediatrics/Nephrology, Burrell College of Osteopathic Medicine, Las Cruces, USA; 3 Nephrology, Rehoboth McKinley Christian Health Care Services, Gallup, USA

**Keywords:** kidney transplant, end stage renal disease (esrd), chronic kidney disease (ckd), rural medicine, hemodialysis, arteriovenous (av) fistula, angiography, venous angioplasty

## Abstract

A 69-year-old Native American female with a past medical history of end-stage renal disease presented to our rural outpatient dialysis access center. One and a half years prior, the patient’s arteriovenous fistula was banded due to venous steal syndrome and now demonstrated an abnormal bruit with decreased blood flow during dialysis. On arteriogram, she was found to have a 90% narrowing of her previously banded cephalic vein along with stenosis of the arterial anastomosis and subclavian vein. Balloon angioplasty was performed on the subclavian vein stenosis, and the banded cephalic vein was ruptured. However, the arterial anastomosis stenosis was left untreated due to the patient’s previous venous steal syndrome.

## Introduction

An arteriovenous (AV) fistula is a connection surgically made between an artery and vein to allow for high blood flow during hemodialysis. Fistulas may be made in many anatomical locations, and the most common types of fistulae are the radiocephalic fistula, the brachiocephalic fistula, and the brachial artery-to-transposed basilic vein fistula [1]. After a successful maturation of 4-6 weeks [2], the fistula may then be used for hemodialysis in patients with end-stage renal disease (ESRD). Although lifesaving, certain complications may arise from this procedure such as stenotic lesions in hemodynamically stressed regions of the vasculature [1]. Dialysis access steal syndrome (DASS), a type of venous steal syndrome, is one of the most dangerous yet common complications of AV fistula access [2]. DASS occurs when blood flow is diverted from the extremity in favor of the high flow anastomosis. However, it may also occur due to peripheral artery disease proximal or distal to the AV anastomoses, decreasing flow to the AV conduit [2]. DASS may initially present with extremity coolness and paresthesia that can chronically develop into more significant extremity pain [2]. If left untreated, it can even result in fingertip ulcerations, motor and sensory deficits, and tissue loss [2].

Techniques such as proximalization of the arterial inflow (PAI) anastomosis, revision using distal inflow (RUDI), ligation of the anastomosis, distal revascularization, and interval ligation (DRIL), and banding of the outflow vein may be utilized in the correction of venous steal syndrome [2,3]. DRIL is the most preferred technique [3]. However, in rural areas with limited access to vascular surgeons, less involved techniques that can be performed by general surgeons, such as banding, can be utilized. However, banding can produce inconsistent results and is associated with the risk of later failure due to thrombosis of the dialysis access [3].

Here we report about a patient who was initially treated for venous steal syndrome by venous banding. Subsequently, the patient developed complications that resulted in diminished blood flow to her brachiocephalic fistula, impairing her ability to receive adequate hemodialysis. We describe a unique approach to band revision that is feasible in a rural health care setting and at the same time prevents venous steal syndrome recurrence.

## Case presentation

A 69-year-old Native American female presented to our rural interventional nephrologist's office for a one-day history of an abnormal arteriovenous (AV) fistula bruit. The previous day, hemodialysis was hampered by decreased blood flow through the vascular access. There was no reported difficulty in accessing the AV fistula at the outpatient dialysis center and volumetric flows were not reported to the interventional nephrologist.

The patient has a history of ESRD secondary to essential hypertension and type 2 diabetes mellitus as well as a previous non-ST elevation myocardial infarction (NSTEMI), which later contributed to the development of heart failure. The patient’s brachiocephalic AV fistula was created one and a half years ago. The patient had no history of prior subclavian central lines. Two weeks after the fistula’s creation, the patient began experiencing left arm pain that was determined to be DASS. The patient subsequently underwent banding of the cephalic vein by our rural general surgeon which successfully relieved the symptoms of DASS.

The patient currently attends hemodialysis three times a week and has the dialysis prescription of sodium 138mEq/L, potassium 3mEq/L, calcium 2.5mEq/L, and bicarbonate 40mEq/L with a blood flow (QB) of 400 mL/min. Since receiving her fistula, the patient has been compliant with her dialysis, attending all but one treatment in the past year and a half.

On examination at our dialysis access center, the patient’s access arm revealed a high-pitched bruit in the proximal 4cm inflow segment of the fistula. No arm swelling, tenderness, or erythema was noted. The patient was vascularly intact with normal arterial and ulnar pulses. There was no apparent cyanosis of the distal digits.

An angiogram of the fistula was performed under conscious sedation induced by midazolam and fentanyl. Throughout the procedure, the patient’s vital signs and electrocardiogram were monitored. The dialysis access was then cannulated, and the contrast medium was injected in an antegrade direction, evaluating the access, draining veins, and central veins. A retrograde arteriogram was also performed to evaluate the arterial anastomosis.

**Figure 1:**
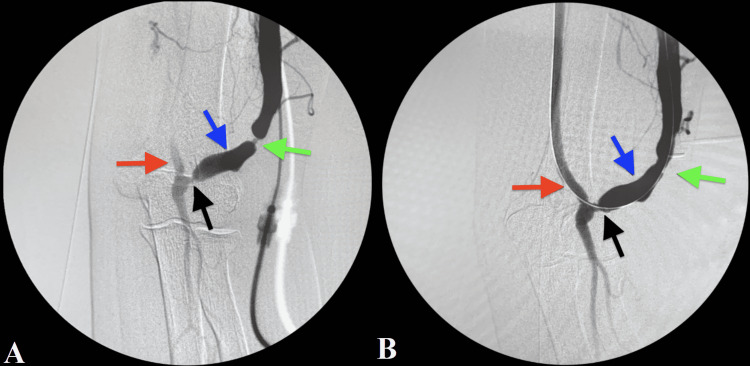
Angiography of arteriovenous fistula with arterial anastomosis stenosis. (A) Pre-band rupture; (B) post-band rupture. Red arrow: brachial artery; Black arrow: arterial anastomosis with approximately 50%-60% stenosis; Blue arrow: cephalic vein; Green arrow: banding with approximately 90% narrowing

**Figure 2:**
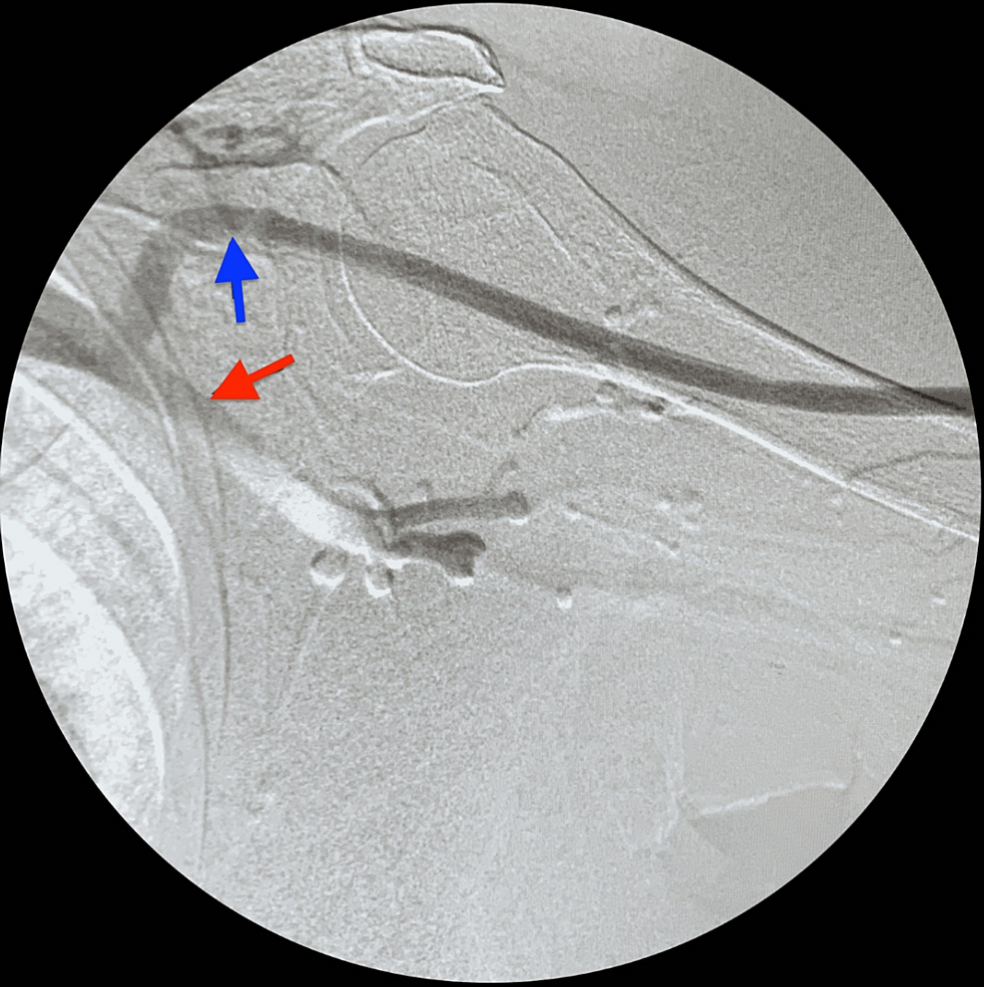
Angiography of proximal cephalic and axillary veins Red arrow: axillary vein; Blue arrow: cephalic vein

**Figure 3:**
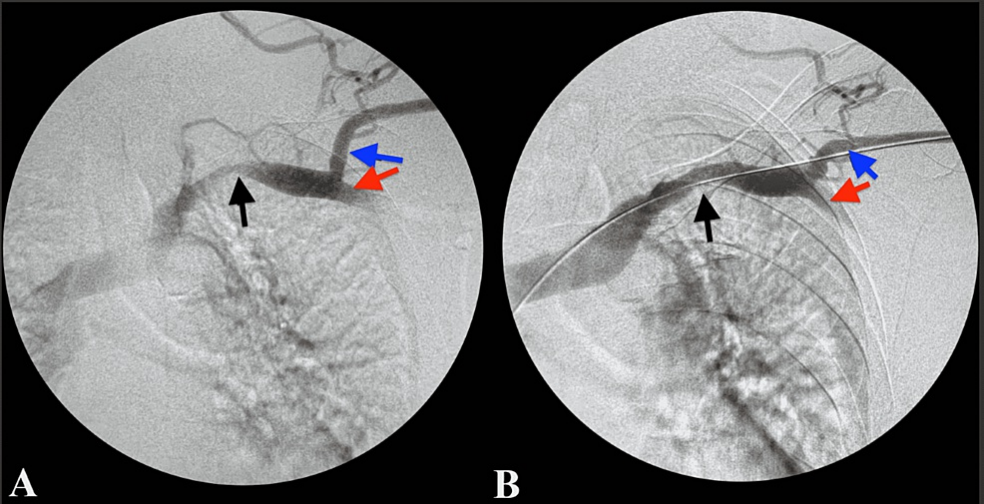
Angiography of subclavian vein stenosis. (A) Pre-angioplasty; (B) post-angioplasty. Black arrow: subclavian vein with approximately 50%-60% stenosis; Blue arrow: cephalic vein; Red arrow: axillary vein

The angiogram (Figures 1A, 1B, 2) showed no stenotic lesion in the access or proximal cephalic vein. However, there was a 50%-60% stenosis in the mid subclavian vein (Figure 3A). A retrograde angiogram (Video 1) shows an area of banding with severe stricture, which significantly limited flow and caused narrowing of about 90% of the cephalic vein. Of note, there was also a 50%-60% arterial anastomotic stenosis (Figure 1A).

**Video 1:** Angiogram of arteriovenous fistula with arterial anastomosis stenosis and banding pre-angioplasty

**Figure 4:**
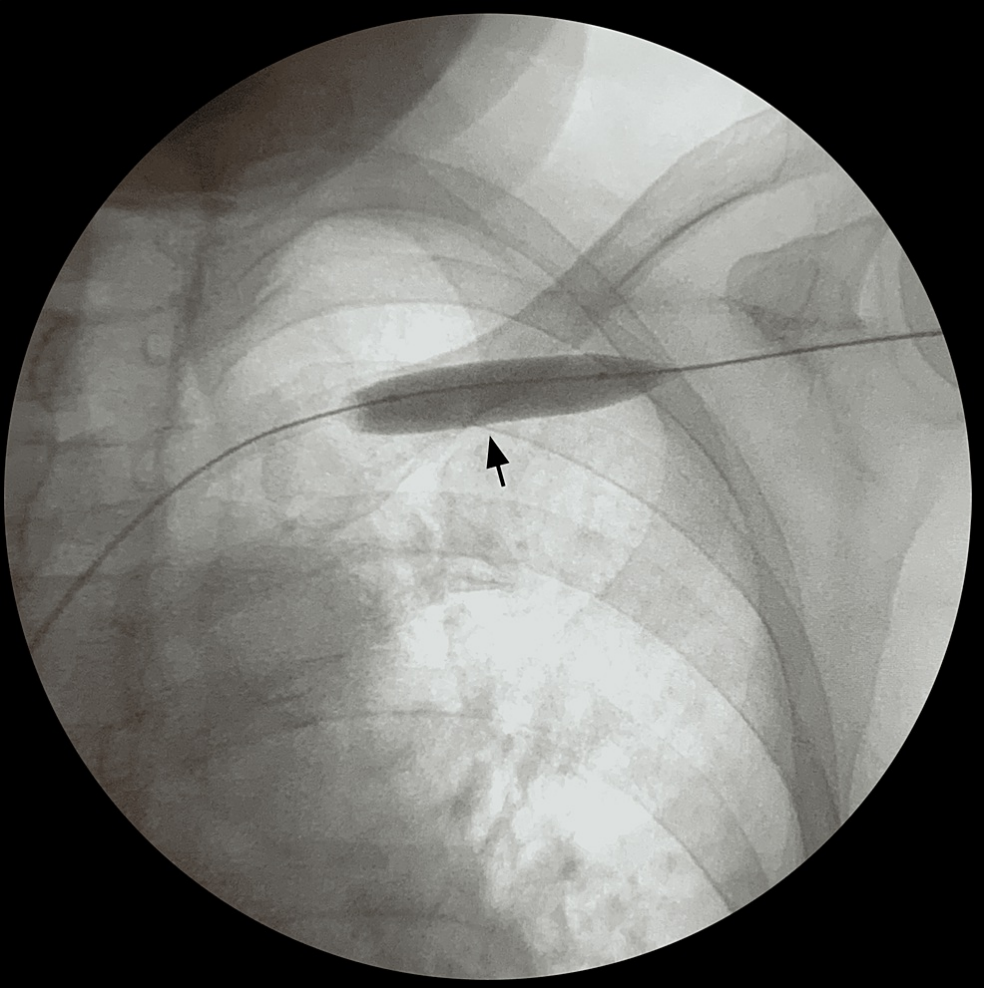
Balloon angioplasty of subclavian vein stenosis Black arrow: Subclavian vein balloon angioplasty

A 0.035-inch guide wire was then advanced into the access. The angiography catheter was then exchanged for an 8F sheath. A 12 x 40mm balloon was advanced over the guidewire towards the stenotic segments of the subclavian vein (Figure 4). A hand syringe assembly was utilized to inflate the balloon, resulting in full effacement of the lesion. Pressure was held for a total of 90 seconds. Follow-up angiogram revealed improvement in flow with less than 50% residual lesion noted (Figure 3B).

Because the juxta-anastomotic banded area and arterial stenosis were not assessable though the antegrade sheath, a second cannulation was performed in a retrograde fashion directed towards the arterial anastomosis using an 18-gauge angiography catheter. A 0.035-inch guidewire was inserted through the angiography catheter into the access and the needle exchanged for a 7F introducer sheath. The banded area affected by severe stricture was ruptured utilizing a conquest 7 x 40 mm balloon with reestablishment of flow noted on follow-up angiogram (Video 2). A stent was prepared and available for the possibility that the band rupture caused development of an aneurysm. However, this potential complication did not occur and there was no need to insert the stent. Given the patient’s history of DASS, a decision was made not to treat the arterial stenosis (Figures 1A, 1B) to prevent recurrence of DASS.

**Video 2:** Balloon rupturing of band

Following the procedure, radial and ulnar pulses were adequate, no cyanosis of the digits was evident and the patient denied distal pain.

A final angiogram was taken at the end of the procedure showing that there was good flow in the access and the draining veins (Video 3). The previously identified areas of stenosis were significantly improved (Figure 1B).

**Video 3:** Angiogram of arteriovenous fistula with arterial anastomosis stenosis post-band rupture

Hemostasis was achieved using a 3-0 nylon suture at the cannulation site, which was to be removed in a few days’ time at the patient’s dialysis center. The patient was discharged from the outpatient interventional nephrologist center within an hour of completion of the procedure and will be followed-up at the dialysis unit for access flows and venous pressures. Signs of venous steal will also be monitored. The patient is scheduled to return to the interventional nephrologist for follow-up in a few months and the purposeful maintenance of the arterial stenosis was explained and noted in the patient’s records.

## Discussion

As of 2020, approximately 558,000 people in the United States are on dialysis [4], with the highest incidence in African Americans, followed by American Indian/Alaska Natives [5]. The reasons for the disproportionate amount of ESRD in these ethnicities are complex. However, socioeconomic disparities are likely to contribute. Especially, in rural settings, these ethnicities face significant barriers to accessing healthcare, including pre-ESRD care and AVF placement [6]. Of note, our patient is of Native American descent and resides in a rural area with limited access to specialized healthcare which may have contributed to the complications that the patient experienced. At the initial presentation with venous steal syndrome, the lack of access to a vascular surgeon resulted in a banding being performed by a general surgeon instead of the more advanced techniques, such as PAI, RUDI, or DRIL that a vascular surgeon may have performed.

Although the general surgeon was skilled in the banding procedure, our patient experienced secondary complications that are well-described for the banding procedure [3]. Banding failure is most common when there is undiagnosed inflow artery stenosis, fistula blood flow is less than the recommended 600-800 mL/min, or when finger pressure is not monitored for flow restriction during the banding procedure [2]. It should be noted that finger pressure was not noted to be monitored during the patient’s initial banding procedure. Perhaps due to the rural setting of the health care center, the necessary equipment and monitoring staff required to measure finger pressure was not available at the time.

The DRIL procedure circumvents the problematic fistula that can induce the venous steal syndrome by creating a bypass from the brachial artery to the ulnar or radial arteries. When compared with banding, DRIL has been noted to result in fewer complications. It allows the original fistula to be preserved and is considered the preferred procedure for the management of DASS in those who can tolerate invasive procedures [3]. Therefore, if our patient had initially been seen in a more resourceful setting with access to a vascular surgeon trained in the DRIL procedure, the band revision described here would probably never have been necessary.

Our patient first experienced acute DASS less than 30 days following her initial AV fistula placement. At that time, her symptoms were typical of acute steal syndrome: pain combined with numbness and tingling sensations [2]. If the initial symptoms would not bring her to the emergency room, further symptoms such as limb coolness, nerve damage, and tissue loss could have progressed into more drastic consequences including loss of the limb [2]. DASS may affect any patient who has a fistula. However diabetic female patients who are older than 60 and have their anastomosis sites at brachial arteries are most at risk for venous steal syndrome [2]. Our patient was therefore at a high risk of developing DASS, as she is diabetic, female, and over 60 years of age. It should also be noted that her fistula was located at her brachial artery instead of her radial artery, which is associated with a 10-fold increase of DASS compared with AV fistulas that use the radial artery [2].

The most common site of stenosis seen in brachiocephalic fistulas is the cephalic arch [1]. However, our patient had two locations of stenosis: the subclavian vein and the proximal site of the fistula anastomosis. Following interventional rupture of the cephalic vein banding, the initial 90% stenosis was converted into a remaining 50%-60% stenotic lesion. Incidentally, this residual stenosis together with the arterial stenosis that was left in place, was just right to limit blood flow enough to prevent recurrence of DASS.

Since the patient underwent complications related to her AVF, her choice to pursue AVF over other modalities such as peritoneal dialysis should also be considered. AVF has a greater risk of failure and complications such as DASS that increases with older age, diabetes and coronary and peripheral artery disease [7]. Compliant patients on hemodialysis attend three dialysis sessions weekly, each of which last approximately three hours. Peritoneal dialysis, however, must be completed daily but can be done in the comfort of the patient’s own home. Although peritoneal dialysis does have an increased risk of infections, there is a greater decrease in mortality compared to those on hemodialysis (20% versus 15%) [4]. Kidney transplantation requiring life-long immunosuppressant therapy is another option for treating ESRD. Kidney transplants are the ideal renal replacement option because it avoids hemodialysis-related complications such as DASS and venous stenosis while also foregoing the daily maintenance required of peritoneal dialysis. However, undergoing a kidney transplant is dependent on organ availability, donor-recipient compatibility, and patient compliance for which socioeconomic and psychosocial factors must be taken into consideration. Deficiencies in awareness of dialysis options and health literacy can also influence a patient’s decision to choose peritoneal dialysis [8]. It is possible that peritoneal dialysis may have been a better option for this patient and resulted in fewer complications, and it is worth noting that peritoneal dialysis is available in our rural setting. However, patient circumstances and concerns could have prevented this option from being initially chosen.

## Conclusions

This case report dramatically demonstrates how the combination of a rural healthcare setting with a lack of access to specialized healthcare providers, such as vascular surgeons, in addition to ethical and socioeconomic determinants of health can impact the healthcare that patients receive and perhaps, more importantly, affect the outcomes of patients. In our case, the patient chose to undergo AVF in lieu of peritoneal dialysis. As a result of her co-morbidities and other risk factors, she ultimately developed DASS. Although she was appropriately treated with the accepted banding procedure, she did not have access to the more advanced DRIL procedure and ultimately developed complications from the banding procedure. A secondary aspect of this patient case report is that sometimes unique and innovative approaches need to be considered when working in rural settings with limited resources. Following the interventional rupture of the stenosis at the side of the banding, the residual 50%-60% stenosis at this site together with the arterial anastomotic fistula stenosis provided just the right amount of blood flow restriction to prevent DASS. Thus, instead of opening the arterial stenosis we conscientiously decided to leave the arterial stenosis untouched. Although only future follow-ups will show if our unique approach indeed prevents the recurrence of DASS in this specific patient, this case report suggests that preventing DASS recurrence can be accomplished even in a resource-limited health care setting.
